# Impact of universal testing and treatment on sexual risk behaviour and herpes simplex virus type 2: a prespecified secondary outcomes analysis of the HPTN 071 (PopART) community-randomised trial

**DOI:** 10.1016/S2352-3018(22)00253-3

**Published:** 2022-11-01

**Authors:** Ethan Wilson, Deborah Donnell, Timothy Skalland, Sian Floyd, Ayana Moore, Nomtha Bell-Mandla, Justin Bwalya, Nkatya Kasese, Rory Dunbar, Kwame Shanaube, Barry Kosloff, Oliver Laeyendecker, Yaw Agyei, Graeme Hoddinott, Peter Bock, Sarah Fidler, Richard Hayes, Helen Ayles, HPTN 071 (PopART) Study Team

**Affiliations:** a Fred Hutchinson Cancer Center, Seattle, WA, USA; b London School of Hygiene and Tropical Medicine, London, UK; c FHI 360, Durham, NC, USA; d Desmond Tutu TB Centre, Department of Paediatrics and Child Health, Faculty of Medicine and Health Sciences, Stellenbosch University, Cape Town, South Africa; e Zambart, Lusaka, Zambia; f Division of Intramural Research, National Institute of Allergy and Infectious Diseases, National Institutes of Health, Bethesda, MD, USA; g Johns Hopkins Univiersity School of Medicine, Baltimore, MD, USA; h Imperial College London, London, UK; i NIHR Imperial Biomedical Research Centre, London, UK

## Abstract

**Background:**

Comprehensive HIV prevention strategies have raised concerns that knowledge of interventions to reduce risk of HIV infection might mitigate an individual's perception of risk, resulting in riskier sexual behaviour. We investigated the prespecified secondary outcomes of the HPTN 071 (PopART) trial to determine whether a combination HIV prevention strategy, including universal HIV testing and treatment, changed sexual behaviour; specifically, we investigated whether there was evidence of sexual risk compensation.

**Methods:**

HPTN 071 (PopART) was a cluster-randomised trial conducted during 2013–18, in which we randomly assigned 21 communities with high HIV prevalence in Zambia and South Africa (total population, approximately 1 million) to combination prevention intervention with universal antiretroviral therapy (ART; arm A), prevention intervention with ART provided according to local guidelines (universal since 2016; arm B), or standard of care (arm C). The trial included a population cohort of approximately 2000 randomly selected adults (aged 18–44 years) in each community (N=38 474 at baseline) who were followed up for 36 months. A prespecified secondary objective was to evaluate the impact of the PopART intervention compared with standard of care on herpes simplex virus type 2 (HSV-2) and sexual behaviour (N=20 422 completed final visit). Secondary endpoints included differences in sexual risk behaviour measures at 36 months and were assessed using a two-stage method for matched cluster-randomised trials. This trial is registered with ClinicalTrials. gov, number NCT01900977.

**Findings:**

The PopART intervention did not substantially change probability of self-reported multiple sex partners, sexual debut, or pregnancy in women at 36 months. Adjusted for baseline community prevalence, reported condomless sex was significantly lower in arm A versus arm C (adjusted prevalence ratio 0·80 [95% CI 0·64–0·99]; p=0·04) but not in arm B versus arm C (0·94 [0·76–1·17]; p=0·55). 3-year HSV-2 incidence was reduced in arm B versus arm C (adjusted risk ratio 0·76 [95% CI 0·63–0·92]; p=0·010); no significant change was shown between arm A versus arm C (0·89 [0·73–1·08]; p=0·199).

**Interpretation:**

We found little evidence of any change in sexual behaviour owing to the PopART interventions, and reassuringly for public health, we saw no evidence of sexual risk compensation. The findings do not help to explain the differences between the two intervention groups of the HPTN 071 (PopART) trial.

**Funding:**

National Institute of Allergy and Infectious Diseases, the National Institutes of Health, the International Initiative for Impact Evaluation (3ie), the Bill & Melinda Gates Foundation, the US President's Emergency Plan for AIDS Relief, and the Medical Research Council UK.

## Introduction

The incidence of HIV in Zambia and South Africa remains high, with an estimated 6·0 per 1000 population aged 15–49 years for Zambia and 6·9 per 1000 population aged 15–49 years for South Africa reported in 2019.^1^ HIV prevention strategies are of crucial importance and need to include a combination of behavioural and biomedical approaches. In the HPTN 071 (PopART) trial,^2^ we evaluated a combination HIV prevention strategy, including universal testing and treatment for HIV, to reduce HIV incidence at population level in Zambia and South Africa between 2013 and 2018. Although the primary focus of the HPTN 071 (PopART) trial was to measure the overall effect of a universal HIV testing and treatment package on HIV incidence, the household-based delivery of HIV testing formed the foundation of the intervention to deliver sexual reproductive health interventions to all households, including counselling, provision of condoms and lubricant, as well as screening and referrals for sexually transmitted infection symptoms.^3^ The aforementioned PopART intervention was delivered in two study arms, one (arm A) implemented universal antiretroviral therapy (ART) from the beginning of the intervention, and the other (arm B) implemented ART according to local guidelines. The standard-of-care arm (arm C) did not implement the PopART intervention, but applied HIV testing and ART according to local guidelines. The HPTN 071 (PopART) trial reported a reduction in HIV incidence of about 20% in the combined intervention arms, compared with the standard of care. However, in single-arm analyses, HIV incidence was significantly reduced in arm B compared with the standard of care, but not when comparing the more intensive intervention arm A with the standard of care.^2^

**Research in context**

**Evidence before this study**

A comprehensive literature review was done before the HPTN 071 (PopART) trial and included the following topics: the global burden of HIV; HIV prevention interventions, including combination of behavioural and biomedical approaches; universal testing and treatment; and HIV incidence. There were no trials of population-level universal testing and treatment or their effects on sexual risk compensation at that time, hence the need to include this as a secondary endpoint of the trial. For the purpose of this analysis, we conducted a rapid literature review of English articles using the terms (“sexual risk” OR “sexual behaviour” OR “risk compensation” OR “behavioural disinhibition”) AND (“hiv”[MESH terms] OR “hiv”[All fields]) from Jan 1, 2000 (when antiretroviral therapy [ART] and biomedical prevention strategies were available), to Jan 13, 2021 (date search was completed), in three electronic databases (PubMed, Embase, and MEDLINE), and included studies among different groups (people who were HIV-negative and people living with HIV) and across several geographical areas (Europe, Australia, USA, and sub-Saharan Africa), including various prevention strategies (male circumcision, microbicides, pre-exposure prophylaxis use, vaccine trials, ART treatment, and HIV testing services) and a range of sexual risk metrics (condomless last sex and number of sexual partners). We identified a total of 120 papers that included measures of sexual risk behaviour or risk compensation related to different HIV prevention strategies, mostly related to male circumcision or pre-exposure prophylaxis, with 25 papers related to HIV testing services and antiretroviral usage. We identified three systematic reviews and meta-analyses of risk compensation, at both individual level and community level, related to HIV testing services or access to antiretrovirals, or both. The studies and reviews reported mixed results. At the individual level, especially in data from Europe and North America, there was some evidence to suggest that the belief that ART reduces the transmissibility of HIV might increase sexual risk behaviour in high-risk groups, but with limited evidence of risk compensation in people living with HIV who are receiving ART, especially in studies from Africa. At the population level, a meta-analysis that explored trends in behaviour after widescale roll-out of ART, including data from demographic and health surveys, found that although condom use was reported to increase, so too was the report of multiple sexual partners.

**Added value of this study**

This study provides novel insight about the population-level effects of a combination HIV prevention strategy that implements universal testing and treatment. We observed no sexual risk compensation and, conversely, no evidence of reduced sexual risk-taking behaviours. We included herpes simplex virus type 2 (HSV-2) incidence as a biological proxy for sexual risk taking and observed a striking similarity between community-level HSV-2 and HIV acquisition.

**Implications of all the available evidence**

Our findings simultaneously show that community-implemented HIV prevention strategies need to identify additional methods to reduce sexual risk, but that risk compensation associated with such interventions was not a cause for concern, as has been previously hypothesised. Our observations about new HSV-2 infections and HIV incidence suggest a need for further investigation of the nature of the relationship between these viruses.

For many years, there has been concern that widespread scale-up of HIV prevention strategies could result in sexual risk compensation, in which knowledge of interventions to reduce risk of HIV infection might cause a change in an individual's perception of risk, and thus result in riskier sexual behaviour.^4^, ^5^ This concern has been postulated for medical male circumcision,^6^, ^7^ microbicides,^5^ pre-exposure prophylaxis (PrEP),^8^, ^9^, ^10^, ^11^ and vaccines.^12^, ^13^, ^14^ Similarly, there has been concern that as HIV testing becomes normalised and the use of ART increases, sexual risk behaviour will increase.^4^ Previous meta-analyses of individual-level risk compensation have looked at ART use and beliefs in Europe, Australia, and the USA after wide-scale ART roll-out^15^ and, more recently, in sub-Saharan Africa.^16^ These studies reported mixed results, with some evidence that the belief that ART reduces transmissibility of HIV might increase sexual risk behaviour in groups at high risk, but with limited evidence of risk compensation in people living with HIV. At the population level, a meta-analysis^17^ using demographic and health survey data across 18 countries during a period of rapid ART scale-up (2004–15) reported that, while condom use was reported to increase, so too was the report of multiple sexual partners.

Here, we address a secondary objective of the HPTN 071 (PopART) trial protocol by examining self-reported sexual risk behaviour data and herpes simplex type 2 (HSV-2) incidence (as a biological proxy of higher risk sexual behaviour) to identify whether the PopART intervention (which included counselling, HIV testing, provison of condoms, and sexually transmitted infection screening) led to a reduction in community-level sexual risk behaviour and if there was sexual risk compensation owing to increased coverage of ART and perception of lower risk. We also assessed whether differences in sexual behaviour help to explain the unexpected findings of reduced effectiveness of the full intervention (arm A) compared with partial intervention (arm B).

## Methods

### Study design and participants

The HPTN 071 (PopART) trial was a three-arm cluster-randomised trial conducted during 2013–18, for which details have been described previously.^3^ Briefly, the trial was conducted in 21 communities in Zambia (n=12) and South Africa (n=9), encompassing a total population of about 1 million. Communities were matched into seven triplets and randomly assigned into one of three arms (arm A, arm B, and arm C) in each triplet. The HPTN 071 (PopART) trial received ethical approval from the University of Zambia (Lusaka, Zambia), Stellenbosch University (Stellenbosch, South Africa), and London School of Hygiene and Tropical Medicine (London, UK). The PopART intervention was delivered to all adult (aged >18 years) members of the 21 communities. In selection of the population cohort, the only inclusion criteria was age (18–44 years). All participants in the population cohort provided written informed consent for their participation in the research. The study protocol is available online.

Here, we report a prespecified secondary outcomes analysis of the HPTN 071 (PopART) trial.

### Randomisation and masking

Communities, which included all members within (approximately 1 million people), were randomly assigned to one of the three arms. The randomisation process for this trial has been described previously;^3^ briefly, we stratified randomisation by country and in each of seven triplets, matched based on geographical location and estimated HIV prevalence. There was no masking of participants or research teams.

### Procedures

Two intervention arms (arm A and arm B) received a combination prevention package (ie, the PopART intervention) that was delivered by specially trained community HIV-care providers (CHiPs). CHiP teams visited all households annually to deliver HIV testing and linkage to care for those identified as HIV-positive. In addition to testing and treatment, at annual visits, households in the intervention arms A and B received HIV education and prevention information (including information on ART as prevention), referral to medical male circumcision services for men who were HIV-negative, prevention of mother-to-child transmission services for pregnant women who were HIV-positive, provision of condoms and lubricant, and sexually transmitted infection and tuberculosis screening and treatment referral. These services were offered to everyone aged 18 years and older in the enrolment year, and to individuals aged 15 years and older in the subsequent rounds. Intervention arm A and arm B were distinct from one another in the strategy for delivering ART via local government clinics; individuals in arm A received universal ART regardless of CD4 cell count from the start of the trial, whereas those in arm B received ART according to local guidelines. The intervention arms were compared with a standard of care arm (arm C), in which participants did not receive the PopART intervention but received HIV testing and ART according to local guidelines, which shifted to universal ART from April, 2016, onward in Zambia and

October, 2016, onward in South Africa.

The effect of the PopART intervention was assessed through the enrolment of a population cohort of approximately 2300 randomly selected adults (age 18–44 years) from each of the 21 study communities. Our analysis population included the cohort of 38 474 enrolled at study baseline (an additional 9827 participants were enrolled in months 12–24, but these individuals are not included in the present analyses). Participants were followed up annually for up to 36 months. Survey questionnaires were administered at each visit, and included demographic characteristics as well as self-reported sexual risk behaviour metrics, using well validated questions, such as are used in demographic and health surveys: whether or not participants have ever had sex, number of sexual partners in the past 12 months, condom use at last sex in the past 12 months, and current pregnancy status (only asked at follow-up rounds). Laboratory-confirmed HSV-2 status was determined for all consenting participants at baseline, and again at the final study visit (month 36) among those who were HSV-2-negative at baseline. HSV-2 was measured using the Kalon HSV-2 assay (Kalon Biological, Guildford, UK) in laboratories in Zambia and South Africa with confirmatory testing of positive, indeterminate, and seroincident samples at the HPTN Laboratory Center in Baltimore, MD, USA. Laboratory-based HIV testing was performed for all participants at all visits. Central laboratories in South Africa and Zambia performed a single fourth-generation HIV test (Abbott Architect HIV Ag/Ab Combo Assay [Abbott, Abbott Park, IL, USA**]**). The HPTN Laboratory Center performed additional testing to confirm all seroincident HIV test results. Rapid HIV testing was offered to all participants in the population cohort annually.

### Outcomes

**Figure 1:**
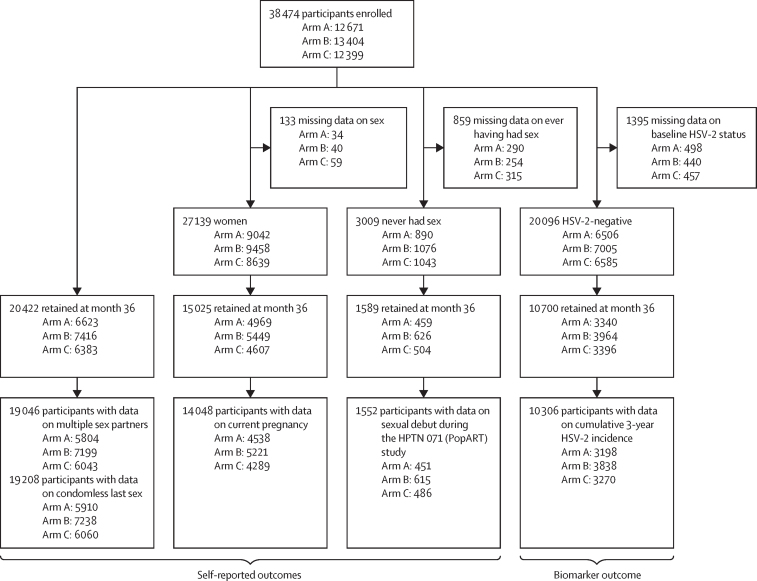
Enrolment and follow-up of outcome-specific cohorts Arm A=combination prevention intervention with universal antiretroviral therapy. Arm B= combination prevention intervention with antiretroviral therapy provided according to local guidelines (universal since 2016). Arm C=standard of care. HSV-2=herpes simplex virus type 2.

Our sexual behaviour metrics led to the following secondary outcomes of the trial with specific cohorts among the 38 474 participants in the population cohort who enrolled at study baseline: multiple sex partners (defined as zero to one partner *vs* more than one, in the past year) and condomless last sex (defined as no condom use at last sex act, in the past year) were analysed among all participants; current pregnancy status was analysed among female individuals; sexual debut was analysed among participants self-reporting never having had sex at baseline; and HSV-2 incidence (cumulative over 3 years) was analysed among participants who were HSV-2-negative at baseline (figure 1). Baseline demographics were summarised by arm to assess balance among the participants on the basis of month 36 retention. For all outcomes, we calculated the community-level proportions with the outcome at month 36, and tabulated the mean proportions by arm. For multiple sex partners and condomless last sex, we calculated the community-level proportions at baseline and the relative change from baseline. We also calculated the community prevalence of HSV-2 at baseline. For HSV-2, the proportion of individuals who were HSV-2-positive at month 36 among participants who were HSV-2-negative at enrolment is interpretable as the cumulative 3-year HSV-2 incidence, or risk.

### Statistical analysis

Sample size calculations were specific to the primary outcomes of the HPTN 071 (PopART) study, and have been described previously.^2^ To test whether the intervention caused a change in sexual risk behaviour, we compared community-level proportions of each outcome at month 36. To test for community-level effect by arm, we used a two-stage statistical approach for the matched-triplet cluster-level outcomes.^18^, ^19^ In the first stage, we fit a logistic regression model for sexual behaviour, including baseline community-level prevalence of the sexual behaviour (when applicable and available), age at baseline (five categories: 18–24 years, 25–29 years, 30–34 years, 35–39 years, and 40–44 years), sex, baseline age and sex interaction, and study triplet. In the second stage, we modelled the log ratio-residuals from the first stage (ie, the ratio of the community-aggregated observed number of events and the community-aggregated expected number of events as predicted by the logistic regression model) using two-way analysis of variance (ANOVA), including triplet and arm as explanatory variables. Within a given triplet, the adjusted prevalence ratio (PR) or adjusted risk ratio (RR) for cumulative HSV-2 incidence, comparing two arms (arm A and arm C) is calculated as the ratio-residuals of the community belonging in arm A, divided by that of arm C. Point estimates of the intervention effect comparing arm A and arm C were calculated as the geometric mean of the adjusted PR, including all seven triplets. If a community-level baseline adjustment was made in stage one, the ANOVA residual degrees of freedom were reduced by 1.

Subgroup analyses were conducted separately among male, female, younger (18–24 years at baseline), older (25–44 years at baseline), baseline HIV-negative, and baseline HIV-positive participants, using the same two-stage method (age and sex were not adjusted for in the age-based and sex-based subgroups, respectively). For each subgroup analysis, the baseline community-level prevalence adjustment was restricted to that subgroup. For HSV-2 analyses, because of the smaller sample size, we adjusted for the overall baseline HSV-2 prevalence. Analyses in which one or more communities did not have at least five participants with data on the outcome of interest were excluded because of inadequate sample size for the cluster-level outcome.

We included interaction tests for evidence of effect modification in cases for which subgroup analyses yielded significant effects by arm. We calculated the difference in log ratio-residuals from the first stage of the main analysis, between binary levels defined by the subgroups (ie, participants who were male and female, younger and older at baseline, and HIV-negative and HIV-positive at baseline). Using the same two-way ANOVA as in the second stage of the main analysis, we modelled the difference in log ratio-residuals defined by a given subgroup, using triplet and arm as explanatory variables, and adjusted the residual degrees of freedom as aformentioned.

All statistical analyses were done using software SAS version 9.4. Barplots were created using R version 4.0.2. p<0·05 was considered statistically significant. During the trial, data were monitored every 6 months by the data and safety monitoring board. This study was registered on ClinicalTrials.gov number, NCT01900977.

### Role of the funding source

The funders of the study had no role in study design, data collection, data analysis, data interpretation, or writing of the report. The trial sponsor, the National Institutes of Health, reviewed and approved the original design of the HPTN 071 (PopART) study and all changes to the study protocol.

## Results

Enrolment and follow-up of outcome-specific cohorts are shown in figure 1. A total of 38 474 participants were enrolled in the population cohort at study baseline between Nov 28, 2013, and March 31, 2015, of whom 27 139 (71%) were women, 3009 (8%) self-reported never having had sex, and 20 096 (54%) were HSV-2-negative. Retention at month 36 was 20 422 (53%) overall (with follow-up occurring between Sept 8, 2017, and July 8, 2018), 15 025 (55%) among women, 1589 (53%) among self-reported never having sex at baseline, and 10 700 (53%) among HSV-2-negative at baseline (figure 1). The cohort at the month 36 analysis was mostly women (15 025 [74%]), aged 25–44 years (12 942 [63%]), HIV-negative (15 775 [79%]), fully or partially completed secondary school (15 214 [76%]), unemployed (14 964 [74%]), and Christian (18 541 [91%]; table 1). More participants were never married (9922 [49%]) than were married or living as married (8690 [43%]), with fewer participants reporting being divorced, separated, or widowed (1627 [8%]). Baseline characteristics were well balanced across arms, although employment was lowest in arm C (1401 [22%]) compared with arm A (1642 [25%]), and arm B had the highest employment rate (2260 [31%]). Retention was higher in women (15 025 [55%]) versus men (5396 [48%]), in those whose baseline age was 25–44 years (12 942 [56%]) versus 18–24 years (7477 [49%]), and in those married or living as married (8690 [57%]) versus never married (9922 [50%]), all with similar retention patterns by arm. We observed balanced retention in all other baseline characteristics presented, including sexual risk behaviours: multiple sex partners and condomless last sex (table 1).

**Table 1:** Baseline characteristics of all participants retained and not retained at month 36

		**Retained at 36 month follow-up**				**Not retained**			
		Arm A (n= 6623)	Arm B (n= 7416)	Arm C (n= 6383)	Total (n= 20 422)	Arm A (n= 6048)	Arm B (n= 5988)	Arm C (n= 6016)	Total (n= 18 052)
Sex									
	Male	1654 (25%)	1967 (27%)	1775 (28%)	5396 (26%)	1941 (32%)	1939 (33%)	1926 (32%)	5806 (32%)
	Female	4969 (75%)	5449 (73%)	4607 (72%)	15 025 (74%)	4073 (68%)	4009 (67%)	4032 (68%)	12 114 (68%)
Age									
	18–24 years	2452 (37%)	2718 (37%)	2307 (36%)	7477 (37%)	2613 (43%)	2461 (41%)	2674 (45%)	7748 (43%)
	25–44 years	4170 (63%)	4698 (63%)	4074 (64%)	12 942 (63%)	3401 (57%)	3487 (59%)	3281 (55%)	10 169 (57%)
HIV status at baseline									
	Negative	5086 (80%)	5811 (80%)	4878 (78%)	15 775 (79%)	4508 (78%)	4424 (77%)	4423 (77%)	13 355 (77%)
	Positive	1299 (20%)	1437 (20%)	1360 (22%)	4096 (21%)	1284 (22%)	1297 (23%)	1327 (23%)	3908 (23%)
Marital status									
	Married or living as if married	2997 (45%)	3075 (42%)	2618 (42%)	8690 (43%)	2366 (40%)	2135 (36%)	2075 (35%)	6576 (37%)
	Never married	3112 (47%)	3623 (49%)	3187 (51%)	9922 (49%)	3180 (53%)	3300 (56%)	3457 (59%)	9937 (56%)
	Divorced, separated, or widowed	487 (7%)	640 (9%)	500 (8%)	1627 (8%)	418 (7%)	460 (8%)	362 (6%)	1240 (7%)
Educational attainment									
	None or primary school only	1377 (21%)	1401 (19%)	1056 (17%)	3834 (19%)	1219 (21%)	1038 (18%)	898 (15%)	3155 (18%)
	Partial or full secondary school	4765 (73%)	5603 (77%)	4846 (77%)	15 214 (76%)	4305 (74%)	4482 (76%)	4593 (78%)	13 380 (76%)
	College or university	391 (6%)	320 (4%)	384 (6%)	1095 (5%)	320 (5%)	351 (6%)	401 (7%)	1072 (6%)
Employment									
	Not employed	4960 (75%)	5088 (69%)	4916 (78%)	14 964 (74%)	4531 (76%)	4246 (72%)	4627 (78%)	13 404 (75%)
	Employed	1642 (25%)	2260 (31%)	1401 (22%)	5303 (26%)	1446 (24%)	1656 (28%)	1284 (22%)	4386 (25%)
Religious affiliation									
	Christian, other	2354 (36%)	2466 (34%)	1977 (31%)	6797 (34%)	2070 (35%)	2056 (35%)	1871 (32%)	5997 (34%)
	Christian, Evangelical (new protestants)	1825 (28%)	1747 (24%)	1540 (24%)	5112 (25%)	1640 (28%)	1362 (23%)	1342 (23%)	4344 (25%)
	Christian, Roman Catholic	816 (12%)	968 (13%)	886 (14%)	2670 (13%)	661 (11%)	664 (11%)	727 (12%)	2052 (12%)
	Christian, Seventh-day Adventist	394 (6%)	396 (5%)	471 (7%)	1261 (6%)	377 (6%)	396 (7%)	449 (8%)	1222 (7%)
	Christian, Traditional Protestants	552 (8%)	653 (9%)	645 (10%)	1850 (9%)	502 (9%)	514 (9%)	628 (11%)	1644 (9%)
	Christian, Jehovah's witness	260 (4%)	292 (4%)	299 (5%)	851 (4%)	208 (4%)	200 (3%)	250 (4%)	658 (4%)
	No religion	262 (4%)	410 (6%)	336 (5%)	1008 (5%)	319 (5%)	372 (6%)	484 (8%)	1175 (7%)
	Islamic	38 (1%)	119 (2%)	61 (1%)	218 (1%)	26 (<1%)	79 (1%)	42 (1%)	147 (1%)
	Other (non-Christian)	45 (1%)	230 (3%)	96 (2%)	371 (2%)	93 (2%)	189 (3%)	93 (2%)	375 (2%)
Multiple sexual partners (past 12 months)									
	No	5941 (95%)	6660 (94%)	5770 (94%)	18 371 (94%)	5251 (95%)	5269 (92%)	5331 (93%)	15 851 (93%)
	Yes	281 (5%)	444 (6%)	373 (6%)	1098 (6%)	285 (5%)	430 (8%)	430 (7%)	1145 (7%)
Condomless last sex (past 12 months)									
	No	3361 (53%)	4385 (62%)	3562 (59%)	11 308 (58%)	3158 (55%)	3597 (63%)	3510 (61%)	10 265 (60%)
	Yes	2999 (47%)	2698 (38%)	2513 (41%)	8210 (42%)	2551 (45%)	2085 (37%)	2223 (39%)	6859 (40%)

Data are n (%).

**Figure 2:**
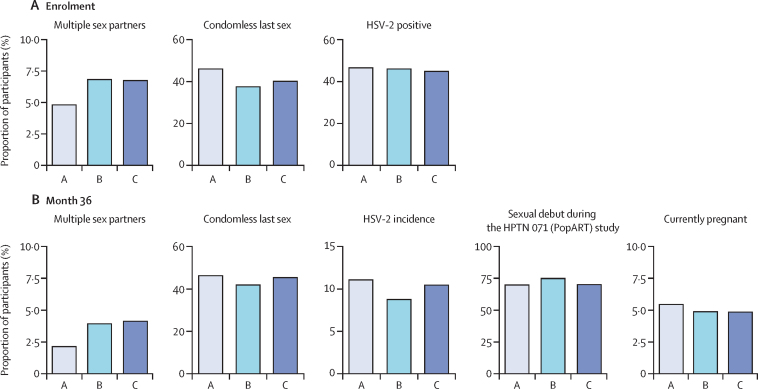
Community-level mean prevalence of HSV-2 and self-reported sexual risk (A) Self-reported multiple sex partners, condomless last sex, and HSV-2-positive status among participants in arm A, arm, B, and arm C at baseline (at enrolment). (B) Self-reported multiple sex partners, condomless last sex, HSV-2 incidence, sexual debut during the HPTN 071 (PopART) study, and current pregnancy among participants in arm A, arm B, and arm C at month 36. HSV-2 incidence is cumulative over 3 years, among participants who were HSV-2 negative at baseline. HSV-2=herpes simplex virus type 2.

At baseline, community-level mean prevalence of self-reported sexual risk was largely balanced across arms, although self-report of multiple sex partners was marginally higher in arm B (7·1%) and arm C (6·7%) than in arm A (4·9%). Condomless last sex was lowest in arm B (38·8%), with higher mean prevalence observed in arm C (40·4%) and arm A (47·3%; figure 2; appendix p 2). At baseline, men (13·8%) were more likely to report having multiple sex partners than women (3·2%). Men (33·0%) were less likely to report condomless sex at the most recent episode than women (45·9%), participants aged 18–24 years (33·6%) reported less condomless sex than participants aged 25 years and older (48·4%), and HIV-positive participants (34·7%) reported less condomless sex than HIV-negative participants (44·2%; appendix p 2).

By month 36, there was a decrease in self-reported multiple sex partners relative to baseline in all trial arms, with the greatest change observed in arm A (60·5% relative decrease in arm A *vs* 41·0% decrease in arm B *vs* 35·0% decrease in arm C; appendix p 2). The observed decrease was highest in women (42·9%, *vs* men [38·9%]), participants aged 25 years and older (51·2%, *vs* participants aged 18–24 years [28·9%]), and participants who were HIV-positive (44·6%, *vs* participants who were HIV-negative [41·6%]). Self-reported condomless last sex decreased by 7·0% in arm A but increased by 7·2% in arm B and 13·1% in arm C. Similar arm-specific directional trends in reported condomless last sex were observed in all subgroups except for the HIV-positive group at baseline, which had the largest observed relative change from baseline. HIV-positive participants at baseline showed the greatest relative decrease at month 36 in self-reported condomless last sex across all study arms, with the largest relative decrease in arm A (42·3%), followed by arm C (29·3%) and arm B (24·7%).

At month 36, self-reported pregnancy was similar across arms (~4·7% women reported to be currently pregnant). Pregnancy proportions were higher among women who were aged 18–24 years (7·3%) and HIV-negative (5·1%), compared with those aged 25 years and older (3·4%) and HIV-positive (3·6%). Among those who said they had never had sex at study baseline, 73–74% reported sexual debut during the HPTN 071 (PopART) study across arms.

HSV-2 prevalence at baseline was around 45% in all arms (figure 2). Higher HSV-2 prevalence was observed in women (53·1%), participants aged 25 years and older (58·3%), and participants who were HIV-positive (84·3%), compared with men (23·8%), participants aged 18–24 years (24·4%), and participants who were HIV-negative (34·1%; appendix p 2). Among participants who were HSV-2-negative at baseline, observed HSV-2 incidence at month 36 was higher in arm A (11·6%) and arm C (11·9%), and lowest in arm B (9·6%). HSV-2 incidence was higher in women (13·8%), participants aged 18–24 years (12·9%), and participants who were HIV-positive (27·6%), compared with men (6·9%), participants aged 25 years and older (9·0%), and participants who were HIV-negative (10·2%).

When adjusted for baseline characteristics, the PopART intervention did not substantially change the likelihood of multiple sex partners, sexual debut during the HPTN 071 (PopART) study, or current pregnancy status (table 2). Reported condomless last sex was significantly lower in arm A compared with arm C (21% reduction; adjusted PR 0·80 [95% CI 0·64–0·99]; p=0·042), but the difference was not significant in arm B compared with arm C (6% reduction; 0·94 [0·76–1·17]; p=0·55). Cumulative 3-year HSV-2 incidence was reduced by 24% in arm B compared with arm C (adjusted RR 0·76 [95% CI 0·63–0·92]; p=0·010); there was little change between arm A and arm C, with only an 11% reduction (0·89 [0·73–1·08]; p=0·22).

**Table 2:** Month 36 self-reported endpoint analysis results

		Participants with endpoint/total participants (community-level mean %)*			**Arm A *vs* arm C**		**Arm B *vs* arm C**	
		Arm A	Arm B	Arm C	Adjusted PR or RR (95% CI)†	p value	Adjusted PR or RR (95% CI)†	p value
**Self-report endpoint**								
More than one sexual partner								
	Overall	118/5804 (1·9%)	290/7199 (4·2%)	251/6043 (4·4%)	PR 0·63 (0·30–1·33)	0·20	PR 1·05 (0·50–2·19)	0·90
Condomless last sex								
	Overall	2681/5910 (44·0%)	2956/7238 (41·6%)	2784/6060 (45·7%)	PR 0·79 (0·64–0·99)	0·04	PR 0·94 (0·75–1·17)	0·53
	Men	555/1453 (36·7%)	691/1919 (35·8%)	680/1674 (40·4%)	PR 0·75 (0·59–0·94)	0·02	PR 0·88 (0·70–1·12)	0·27
	Women	2126/4457 (46·2%)	2265/5319 (43·8%)	2104/4386 (47·9%)	PR 0·81 (0·65–1·01)	0·06	PR 0·96 (0·77–1·19)	0·66
	Aged 18–24 years	992/2262 (39·8%)	950/2659 (36·1%)	909/2214 (40·0%)	PR 0·80 (0·60–1·06)	0·11	PR 0·95 (0·71–1·26)	0·67
	Aged 25–44 years	1689/3648 (46·3%)	2006/4579 (44·9%)	1875/3845 (49·2%)	PR 0·79 (0·65–0·95)	0·02	PR 0·94 (0·78–1·14)	0·50
	HIV-positive	265/1111 (23·3%)	301/1412 (21·7%)	331/1334 (24·6%)	PR 0·84 (0·65–1·09)	0·17	PR 0·97 (0·75–1·25)	0·79
	HIV-negative	2347/4591 (48·7%)	2588/5660 (46·4%)	2382/4589 (51·3%)	PR 0·80 (0·65–0·98)	0·03	PR 0·91 (0·74–1·12)	0·34
Currently pregnant								
	Overall	226/4538 (5·0%)	231/5221 (4·6%)	199/4289 (4·6%)	PR 1·00 (0·79–1·26)	1·00	PR 0·96 (0·76–1·21)	0·68
Sexual debut during the HPTN 071 (PopART) study								
	Overall	315/451 (72·8%)	460/615 (74·1%)	341/486 (73·3%)	PR 1·00 (0·85–1·17)	0·96	PR 1·00 (0·85–1·18)	0·95
**Biomarker endpoint**								
3-year HSV-2 incidence‡								
	Overall	354/3198 (11·6%)	336/3838 (9·6%)	342/3270 (11·9%)	RR 0·89 (0·73–1·08)	0·22	RR 0·76 (0·63–0·93)	0·01
	Men	80/1127 (7·5%)	71/1440 (5·3%)	89/1321 (7·8%)	RR 0·93 (0·63–1·39)	0·71	RR 0·64 (0·43–0·95)	0·03
	Women	274/2071 (14·1%)	265/2398 (12·4%)	253/1948 (14·8%)	RR 0·89 (0·73–1·08)	0·21	RR 0·81 (0·67–0·98)	0·04
	Aged 18–24 years	220/1730 (13·3%)	211/1972 (11·4%)	211/1701 (14·0%)	RR 0·85 (0·65–1·11)	0·21	RR 0·76 (0·58–0·99)	0·04
	Aged 25–44 years	134/1468 (9·7%)	125/1866 (7·5%)	131/1568 (9·7%)	RR 0·95 (0·72–1·26)	0·70	RR 0·77 (0·58–1·03)	0·07
	HIV-positive	38/148 (24·8%)	47/167 (27·4%)	44/142 (30·6%)	RR 0·90 (0·58–1·38)	0·59	RR 0·96 (0·63–1·48)	0·85
	HIV-negative	316/3033 (11·0%)	289/3665 (8·7%)	298/3118 (11·0%)	RR 0·90 (0·72–1·13)	0·32	RR 0·75 (0·60–0·94)	0·02

HSV-2=herpes simplex virus 2. PR=prevalence ratio. RR=risk ratio.

* Average community-level percentage, aggregated by arm.

† Adjusted for baseline age, sex, age and sex interaction, and study triplet. Additionally for outcomes multiple sex partners, condomless last sex, and 3-year HSV-2 incidence, respective baseline community prevalence adjustment was made.

‡ The proportion of new HSV-2 cases among participants who were HSV-2-negative at enrolment is interpretable as cumulative 3-year HSV-2 incidence, with associated adjusted RR, for comparisons by arm.

Differences in new HSV-2 infections within subgroups showed similar results for the whole cohort: comparing arm B and arm C, statistically significant reductions were shown among men (adjusted RR 0·64 [95% CI 0·43–0·95]; p=0·030), women (0·81, 0·67–0·98; p=0·035), participants aged 18–24 years (0·76, 0·58–0·99; p=0·043), and participants who were HIV-negative (0·75 [0·60–0·93]; p=0·015; appendix pp 3, 4). Reported condomless last sex was significantly lower in arm A compared with arm C among men (adjusted PR 0·75 [95% CI 0·60–0·95]; p=0·020), participants aged 25 years and older (0·79, 0·65–0·95; p=0·016), and participants who were HIV-negative (0·80, 0·65–0·98; p=0·034). Among all statistically significant subgroup analyses, none showed evidence of effect modification after testing for arm and subgroup interactions (appendix p 5). Results for subgroups of participants aged 25 years and older and participants who were HIV-positive were omitted for the outcome sexual debut during the HPTN 071 (PopART) study because there were two communities and 15 communities, respectively, with less than five participants contributing to the analyses.

## Discussion

From these analyses we have limited evidence that the PopART intervention led to a change in reported sexual behaviour. We saw a significant reduction in condomless last sex in arm A compared with arm C, but not in arm B compared with arm C. We did not observe any difference as a result of the intervention in the reported number of sexual partners, sexual debuts, or reported pregnancies by arm. Although the PopART intervention was designed to provide combination HIV prevention, which included condom promotion and provision, along with symptomatic screening for sexually transmitted infections, the degree to which this prevention strategy would change individual risk behaviour could be questioned. Many individuals were seen only once per year by the CHiPs, although as the intervention became established many CHiPs reported anecdotally being stopped in the street and asked for additional condoms by their clients.

Similarly, we found no evidence of sexual risk compensation in the intervention arms. Self-reported changes in sexual behaviour could not explain the unexpected trial finding of a reduction in population HIV incidence in arm B compared with arm C (the standard of care), but a smaller reduction in population HIV incidence for arm A compared with arm C.^2^ However, these results do show a striking consistency between HIV incidence and HSV-2 incidence, with HSV-2 incidence mirroring the HIV-incidence efficacy results observed in the HPTN 071 (PopART) trial: in arm B compared with arm C, a 30% reduction was observed in HIV incidence and a 24% reduction in HSV-2 incidence; in arm A versus arm C, we observed a non-significant 7% reduction in HIV incidence and an 11% reduction in HSV-2 incidence.^2^ In this analysis, HSV-2 incidence was used as a proxy for sexual risk. Our cumulative 3-year HSV-2 incidence (1032 of 10 306 participants) was similar to the annual incidence in other studies from sub-Saharan Africa, ranging from 2 to 5 cases per 100 person years.^20^, ^21^ The similar results of HIV and HSV-2 in the trial emphasise the potential importance of shared risk factors for both HSV-2 and HIV, but the little evidence to suggest that the PopART interventions changed sexual risk behaviour might support the argument that HSV-2 infection could have a cofactor effect on HIV acquisition and transmission,^22^, ^23^ despite unsupportive results from trials of the effects of HSV-2 suppression on HIV incidence,^24^, ^25^ and with the caveat that discordant results between biomarker and self-report metrics might be a result of reporting inaccuracies.

In our study, self-report of multiple sexual partners decreased over time in all three study arms, and this decrease might be a result of increased age (by 3 years) or related to retention at 36 months of the cohort. This cohort was aged 18–44 years at baseline, with 37% aged 25 years and younger and in the elapsed 3 years, it is probable that many participants could have married or settled into more stable sexual partnerships, therefore reducing the number of sexual partners but potentially increasing condomless sex. The questions asked of the entire cohort and reported in this paper, although commonly used (eg, in demographic and health surveys) and well validated, were necessarily brief and simplified measures of sexual behaviour and, therefore, did not distinguish between the types of partner, with condom use being reported during last sex. Time trends in sexual behaviour have been evaluated in sub-Saharan Africa after the widescale roll-out of ART. Using data from the Demographic and Health Survey, Shaefer and colleagues^26^ examined repeated cross-sectional surveys from 11 countries and showed that there were widescale reported changes in sexual behaviour over the period of ART scale-up, including increased reporting of multiple and non-regular sexual partners, but that this finding was to some extent balanced by increasing reporting of condom use. Another meta-analysis, including studies of reported sexual behaviour before and after 2004 (a year of increasing access to ART in Africa), reported decreased condomless sex and reporting of multiple partners;^17^ however, demographic and health surveys reported in this meta-analysis showed an increased reporting of condom use but increased reporting of multiple partners. The concept of ART as prevention, and more recently the evidence-based concept that people who have a sustained undetectable HIV viral load cannot sexually transmit HIV—ie, undetectable=untransmittable (U=U)—was largely unknown in communities at the start of the trial,^27^ and although this concept was discussed within the intervention there was no widescale promotion during the trial period.

Although we found some evidence of sexual behaviour change in HIV-negative participants, there was no such evidence among HIV-positive individuals across trial arms. However, individuals who were HIV-positive at baseline reported reduced condomless last sex over time in all arms. A previous large trial of HIV counselling and testing found no reduction in sexual risk behaviour at population level,^28^ except among HIV-positive participants who reported increased condom use. A recent systematic review and meta-analysis of the effect of HIV testing services on sexual behaviour also found limited evidence of sexual behaviour change when comparing either HIV testing services versus no HIV testing services or more intensive versus less intensive HIV testing services.^29^ The study did find some evidence of a reduction in condomless sex after HIV testing services versus before HIV testing services, especially in individuals who tested HIV-positive. In our study, all individuals in the population cohort were offered HIV counselling and testing at each visit, regardless of study arm, and therefore this might have affected our ability to detect a difference in individuals testing positive for HIV by arm.

This study has notable strengths. Assessment of sexual behaviour was based on a large number of randomly selected individuals living in the communities and, therefore, exposed to either intervention or standard of care; as individuals were not selected on the basis of HIV risk, our results are representative of the general population of the communities. Laboratory assessment of HSV-2 acquisition is a novel objective measure of sexual behaviours.

A limitation of our study is that, with the exception of HSV-2, sexual behaviour was self-reported and subject to social-desirability reporting bias in repeated assessments in the population cohort. Given that the intervention did include discussion of sexual risk and provision of condoms, it is possible that these biases could have been differential by trial arm, but the use of a similar methodology of a randomly sampled population cohort who were interviewed by a completely separate research team to the intervention providers (CHiPs) mitigates this somewhat. In this study we report simple indicators of sexual behaviour that could be collected from all members of the population cohort, and so cannot report more nuanced sexual behaviours such as condom use by partner type. Cumulative retention at 36 months was quite low, and there is a risk that by 36 months, representativeness was compromised. However, although our analysis population included more individuals who were female, aged 25–44 years at baseline, and currently married, we found no evidence of differential drop-out across arms by sexual behaviour characteristics.

This study supports that the combination HIV prevention strategy deployed in the HPTN 071 (PopART) trial did not result in sexual risk compensation at the population level. The study also did not show differential behavioural change based on self-reported measures as an explanation for the primary trial outcome, for which HIV incidence was found to have reduced in one of the intervention arms more than the other. Despite delivering universal HIV counselling and testing, along with widespread condom distribution, we saw little evidence of sexual behaviour change. HIV prevention interventions need to identify additional methods to improve condom use and reduce sexual risk. The strikingly similar results for the reduction of HIV and HSV-2 incidence in arm B compared with the standard of care, despite limited evidence of behaviour change, merit further investigation.

For more on **the HPTN 071 (PopART) trial protocol** see https://clinicaltrials.gov/ProvidedDocs/77/NCT01900977/Prot_ICF_000.pdf

## Data sharing

Data used in this study will be available on request immediately after publication, with no end date. This includes de-identified participant data with a data dictionary. A data archive will be held at Fred Hutchinson Cancer Center, Seattle, WA, USA. Requests can be sent to HPTN-Data-Access@scharp.org.

## Declaration of interests

HA and DD report on behalf of the entire study team, grant funding from: the NIH, the international initiative for impact evaluation (3ie), the US President's Emergency Plan for AIDS Relief (PEPFAR), and the Bill & Melinda Gates Foundation (also reflected in Acknowledgments). HA reports membership in the technical review panel for the Global Fund. NB-M is a member on the HPTN Ethics Working Group. All other authors declare no competing interests.
