# Supplementary material for: Impact of universal testing and treatment on sexual risk behaviour and herpes simplex virus type 2: a prespecified secondary outcomes analysis of the HPTN 071 (PopART) community-randomised trial
Source: Lancet HIV. 2022 Nov 1;9(11):e760–70. doi: 10.1016/S2352-3018(22)00253-3 (PMC9646971; doi:10.1016/S2352-3018(22)00253-3)
Supplement: Supplementary appendix [file mmc1.pdf]

THE LANCET
HIV

Supplementary appendix

This appendix formed part of the original submission and has been peer reviewed. We post it as supplied by the authors.

Supplement to: Wilson E, Donnell D, Skalland T, et al. Impact of universal testing and treatment on sexual risk behaviour and herpes simplex virus type 2: a prespecified secondary outcomes analysis of the HPTN 071 (PopART) community-randomised trial. *Lancet HIV* 2022; **9:** e760–70.

Impact of universal testing and treatment on sexual risk behaviour and HSV-2: HPTN 071 (PopART) a community-randomized trial

Appendix

Table of contents

Supplementary Table S1: Baseline and Month 36 prevalence and relative change....2
Supplementary Table S2: All month 36 main and subgroup analysis results....3-4
Supplementary Table S3: All month 36 interaction test results....5
List of HPTN 071 (PopART) study team members....6

Table S1: Baseline and Month 36 prevalence and relative change

		Baseline				Month 36				Relative change			
**Outcome**	**Group**	**Arm A**	**Arm B**	**Arm C**	**All arms**	**Arm A**	**Arm B**	**Arm C**	**All arms**	**Arm A**	**Arm B**	**Arm C**	**All arms**
**Multiple sexual partners**	Overall	4.91%	7.07%	6.71%	6.23%	1.94%	4.17%	4.36%	3.49%	-60.5%	-41.0%	-35.0%	-44.0%
	Men	11.3%	16.4%	13.8%	13.8%	5.22%	8.87%	11.2%	8.43%	-53.8%	-45.9%	-18.8%	-38.9%
	Women	2.39%	3.41%	3.78%	3.19%	1.16%	2.48%	1.82%	1.82%	-51.5%	-27.3%	-51.9%	-42.9%
	18-24	5.23%	7.46%	7.69%	6.79%	2.68%	5.55%	6.25%	4.83%	-48.8%	-25.6%	-18.7%	-28.9%
	25+	4.76%	7.10%	6.20%	6.02%	1.73%	3.58%	3.50%	2.94%	-63.7%	-49.6%	-43.5%	-51.2%
	HIV+	5.09%	5.83%	6.68%	5.87%	2.31%	4.06%	3.37%	3.25%	-54.6%	-30.4%	-49.6%	-44.6%
	HIV-	4.89%	7.55%	7.04%	6.49%	2.01%	4.47%	4.90%	3.79%	-58.9%	-40.8%	-30.4%	-41.6%
**Condomless last sex**	Overall	47.3%	38.8%	40.4%	42.2%	44.0%	41.6%	45.7%	43.8%	-6.98%	7.22%	13.1%	3.79%
	Men	36.6%	31.1%	31.3%	33.0%	36.7%	35.8%	40.4%	37.6%	0.27%	15.0%	29.1%	13.9%
	Women	51.2%	42.2%	44.4%	45.9%	46.2%	43.8%	47.9%	46.0%	-9.77%	3.79%	7.88%	0.22%
	18-24	38.9%	29.8%	32.2%	33.6%	39.8%	36.1%	40.0%	38.6%	2.31%	21.10%	24.2%	14.9%
	25+	53.7%	44.9%	46.6%	48.4%	46.3%	44.9%	49.2%	46.8%	-13.8%	0.0%	5.58%	-3.31%
	HIV+	40.4%	28.8%	34.8%	34.7%	23.3%	21.7%	24.6%	23.2%	-42.3%	-24.7%	-29.3%	-33.1%
	HIV-	48.7%	41.6%	42.2%	44.2%	48.7%	46.4%	51.3%	48.8%	0.00%	11.5%	21.6%	10.4%
**Currently pregnant**	Overall					4.97%	4.60%	4.58%	4.72%				
	18-24					7.56%	6.99%	7.42%	7.32%				
	25+					3.48%	3.40%	3.24%	3.37%				
	HIV+					3.23%	3.58%	4.02%	3.61%				
	HIV-					5.43%	4.91%	4.87%	5.07%				
**Sexual debut during PopART**	Overall					72.8%	74.1%	73.3%	73.4%				
	Men					73.5%	75.6%	74.5%	74.5%				
	Women					71.9%	72.2%	70.8%	71.6%				
	18-24					73.2%	72.9%	75.2%	73.8%				
	25+					70.6%	81.1%	82.2%	78.0%				
	HIV-					72.8%	73.6%	74.3%	73.6%				
**HSV-2[a]**	Overall	45.1%	44.7%	44.1%	44.6%	11.6%	9.55%	11.9%	11.0%				
	Men	24.7%	23.1%	23.5%	23.8%	7.48%	5.25%	7.82%	6.85%				
	Women	53.2%	53.4%	52.8%	53.1%	14.1%	12.4%	14.8%	13.8%				
	18-24	24.8%	24.8%	23.7%	24.4%	13.3%	11.4%	14.0%	12.9%				
	25+	59.0%	57.9%	58.0%	58.3%	9.74%	7.48%	9.71%	8.98%				
	HIV+	84.6%	85.6%	82.7%	84.3%	24.8%	27.4%	30.6%	27.6%				
	HIV-	35.1%	34.1%	33.2%	34.1%	11.0%	8.68%	11.0%	10.2%				

[a]Baseline values describe prevalence, month 36 describes cumulative 3-year incidence among baseline HSV-2 negative

Table S2: All month 36 main and subgroup analysis results

Variable	Arm A	Arm B	Arm C	A vs C		B vs C	
				Adj. PR[b] (95% CI)	P value	Adj. PR (95% CI)	P value
**More than 1 sexual partner**							
Overall	118/5804 (1.94%)	290/7199 (4.17%)	251/6043 (4.36%)	0.63 (0.30, 1.32)	0.20	1.05 (0.50, 2.19)	0.89
Men	75/1409 (5.22%)	163/1903 (8.87%)	174/1662 (11.2%)	0.60 (0.32, 1.11)	0.096	0.74 (0.40, 1.37)	0.30
Women	43/4395 (1.16%)	127/5296 (2.48%)	77/4381 (1.82%)	0.62 (0.21, 1.89)	0.37	1.62 (0.54, 4.93)	0.36
Younger	64/2226 (2.68%)	136/2642 (5.55%)	122/2206 (6.25%)	0.82 (0.50, 1.35)	0.41	1.12 (0.68, 1.84)	0.63
Older	54/3578 (1.73%)	154/4557 (3.58%)	129/3836 (3.50%)	0.49 (0.18, 1.36)	0.15	0.93 (0.34, 2.58)	0.88
HIV+	18/1087 (2.31%)	57/1414 (4.06%)	42/1328 (3.37%)	0.52 (0.18, 1.48)	0.20	1.41 (0.50, 3.99)	0.49
HIV-	95/4517 (2.01%)	228/5621 (4.47%)	206/4579 (4.90%)	0.64 (0.33, 1.24)	0.17	0.98 (0.51, 1.90)	0.95
**Condomless last sex**							
Overall	2681/5910 (44.0%)	2956/7238 (41.6%)	2784/6060 (45.7%)	0.80 (0.64, 0.99)	0.042	0.94 (0.76, 1.17)	0.55
Men	555/1453 (36.7%)	691/1919 (35.8%)	680/1674 (40.4%)	0.75 (0.60, 0.95)	0.020	0.89 (0.70, 1.12)	0.29
Women	2126/4457 (46.2%)	2265/5319 (43.8%)	2104/4386 (47.9%)	0.81 (0.65, 1.01)	0.063	0.96 (0.77, 1.19)	0.67
Younger	992/2262 (39.8%)	950/2659 (36.1%)	909/2214 (40.0%)	0.80 (0.60, 1.06)	0.11	0.95 (0.71, 1.26)	0.67
Older	1689/3648 (46.3%)	2006/4579 (44.9%)	1875/3845 (49.2%)	0.79 (0.65, 0.95)	0.016	0.94 (0.78, 1.14)	0.50
HIV+	265/1111 (23.3%)	301/1412 (21.7%)	331/1334 (24.6%)	0.85 (0.65, 1.10)	0.19	0.97 (0.74, 1.27)	0.81
HIV-	2347/4591 (48.7%)	2588/5660 (46.4%)	2382/4589 (51.3%)	0.80 (0.65, 0.98)	0.034	0.91 (0.75, 1.12)	0.35
**Currently pregnant**							
Overall	226/4538 (4.97%)	231/5221 (4.60%)	199/4289 (4.58%)	1.00 (0.79, 1.26)	0.98	0.96 (0.76, 1.21)	0.70
Younger	126/1565 (7.56%)	118/1720 (6.99%)	102/1341 (7.42%)	0.97 (0.67, 1.41)	0.87	0.94 (0.65, 1.37)	0.74
Older	100/2973 (3.48%)	113/3501 (3.40%)	97/2947 (3.24%)	1.06 (0.78, 1.43)	0.70	1.03 (0.76, 1.39)	0.83
HIV+	35/1024 (3.23%)	45/1225 (3.58%)	44/1134 (4.02%)	0.87 (0.54, 1.41)	0.55	1.02 (0.63, 1.64)	0.93
HIV-	184/3364 (5.43%)	181/3866 (4.91%)	150/3056 (4.87%)	0.99 (0.73, 1.32)	0.91	0.94 (0.70, 1.25)	0.63
**Sexual debut during PopART[a]**							
Overall	315/451 (72.8%)	460/615 (74.1%)	341/486 (73.3%)	0.99 (0.85, 1.17)	0.95	1.00 (0.85, 1.18)	0.96
Men	135/189 (73.5%)	184/249 (75.6%)	175/230 (74.5%)	0.99 (0.80, 1.22)	0.91	1.01 (0.81, 1.24)	0.96
Women	180/262 (71.9%)	276/366 (72.2%)	166/256 (70.8%)	1.05 (0.79, 1.39)	0.71	1.05 (0.79, 1.39)	0.72
Younger	272/392 (73.2%)	362/503 (72.9%)	281/411 (75.2%)	0.99 (0.84, 1.16)	0.87	0.98 (0.83, 1.15)	0.77
HIV-	290/417 (72.8%)	431/581 (73.6%)	320/457 (74.3%)	1.01 (0.53, 1.93)	0.96	1.04 (0.55, 1.98)	0.89
**HSV-2 incidence (3 yr.)**							
Overall	354/3198 (11.6%)	336/3838 (9.55%)	342/3270 (11.9%)	0.89 (0.73, 1.08)	0.20	0.76 (0.63, 0.92)	0.010
Men	80/1127 (7.48%)	71/1440 (5.25%)	89/1321 (7.82%)	0.93 (0.63, 1.38)	0.70	0.64 (0.43, 0.95)	0.030
Women	274/2071 (14.1%)	265/2398 (12.4%)	253/1948 (14.8%)	0.89 (0.73, 1.07)	0.19	0.81 (0.67, 0.98)	0.035
Younger	220/1730 (13.3%)	211/1972 (11.4%)	211/1701 (14.0%)	0.85 (0.65, 1.11)	0.21	0.76 (0.58, 0.99)	0.043

Older	134/1468 (9.74%)	125/1866 (7.48%)	131/1568 (9.71%)	0.95 (0.72, 1.26)	0.70	0.77 (0.58, 1.03)	0.071
HIV+	38/148 (24.8%)	47/167 (27.4%)	44/142 (30.6%)	0.91 (0.60, 1.39)	0.64	0.93 (0.61, 1.42)	0.72
HIV-	316/3033 (11.0%)	289/3665 (8.68%)	298/3118 (11.0%)	0.90 (0.72, 1.12)	0.30	0.75 (0.60, 0.93)	0.015

[a] Older and HIV-positive subgroups were omitted as there were 2 and 15 communities respectively with less than 5 participants contributing to the analyses.

[b] Adjusted prevalence ratio. For HSV-2 this is interpretable as an adjusted risk ratio (Adj RR). Adjusted for baseline age, sex and study triplet (age and sex were not adjusted for in the sex- and age-based subgroups, respectively). Additionally for outcomes: multiple sex partners, condomless last sex, and HSV-2 incidence (3 yr.), baseline community prevalence adjustment of the respective outcomes was made. For each subgroup analysis, the baseline community-level prevalence adjustment was restricted to that subgroup, except for HSV-2 analyses due to smaller sample size, adjusted for the overall baseline HSV-2 prevalence.

Table S3: All month 36 interaction test results

Variable	Interaction tested	Comparison	PR[a] (95% CI)	P value
**More than 1 sexual partner**	Age	A vs C	1.56 (0.86, 2.84)	0.13
		B vs C	1.07 (0.59, 1.95)	0.80
	Sex	A vs C	0.96 (0.42, 2.22)	0.92
		B vs C	0.57 (0.25, 1.31)	0.16
	Baseline HIV status	A vs C	1.14 (0.56, 2.34)	0.69
		B vs C	0.81 (0.40, 1.66)	0.54
**Condomless last sex**	Age	A vs C	1.00 (0.87, 1.16)	0.94
		B vs C	1.00 (0.86, 1.15)	0.97
	Sex	A vs C	0.92 (0.78, 1.08)	0.27
		B vs C	0.96 (0.82, 1.13)	0.62
	Baseline HIV status	A vs C	0.98 (0.83, 1.15)	0.75
		B vs C	1.01 (0.86, 1.19)	0.85
**Currently pregnant**	Age	A vs C	0.96 (0.59, 1.58)	0.87
		B vs C	0.92 (0.56, 1.52)	0.74
	Baseline HIV status	A vs C	1.13 (0.62, 2.07)	0.66
		B vs C	0.95 (0.52, 1.75)	0.87
**Sexual debut during PopART[b]**	Sex	A vs C	0.94 (0.67, 1.33)	0.71
		B vs C	0.96 (0.68, 1.36)	0.80
**HSV-2 incidence (3 yr.)**	Age	A vs C	0.92 (0.62, 1.36)	0.65
		B vs C	0.99 (0.67, 1.46)	0.96
	Sex	A vs C	1.07 (0.72, 1.59)	0.70
		B vs C	0.80 (0.54, 1.18)	0.23
	Baseline HIV status	A vs C	1.05 (0.64, 1.75)	0.83
		B vs C	0.82 (0.49, 1.36)	0.41

[a] Prevalence ratio. For HSV-2 this is interpretable as a risk ratio (RR).
[b]Excluded age and baseline HIV status as older and HIV-positive at baseline groups were excluded from the subgroup analyses.

The HPTN 071 (PopART) Study Team:

Richard Hayes (London School of Hygiene & Tropical Medicine, UK), Sarah Fidler (Imperial College, UK), Nulda Beyers (University of Stellenbosch, South Africa), Helen Ayles (Zambart, Zambia; and London School of Hygiene & Tropical Medicine, UK), Peter Bock (University of Stellenbosch, South Africa), Wafaa El-Sadr (HIV Prevention Trials Network (HPTN) Leadership and Operations Centre, USA), Myron Cohen (HIV Prevention Trials Network (HPTN) Leadership and Operations Centre, USA), Susan Eshleman (HPTN Laboratory Centre, Johns Hopkins University, USA), Yaw Agyei (HPTN Laboratory Centre, Johns Hopkins University, USA), Estelle Piwowar-Manning (HPTN Laboratory Centre, Johns Hopkins University, USA), Virginia Bond (Zambart, Zambia; and London School of Hygiene & Tropical Medicine, UK), Graeme Hoddinott (University of Stellenbosch, South Africa), Deborah Donnell (HPTN Statistical and Data management centre (SDMC), USA), Sian Floyd (London School of Hygiene & Tropical Medicine, UK), Ethan Wilson (HPTN Statistical and Data management centre (SDMC), USA), Lynda Emel (HPTN Statistical and Data management centre (SDMC), USA), Heather Noble (HPTN Statistical and Data management centre (SDMC), USA), David Macleod (London School of Hygiene & Tropical Medicine, UK), David Burns (NIAID, USA), Christophe Fraser (University of Oxford, UK), Anne Cori (Imperial College, UK), Nirupama Sista (HIV Prevention Trials Network (HPTN) Leadership and Operations Centre, USA), Sam Griffith (HIV Prevention Trials Network (HPTN) Leadership and Operations Centre, USA), Ayana Moore (HIV Prevention Trials Network (HPTN) Leadership and Operations Centre, USA), Tanette Headen (HIV Prevention Trials Network (HPTN) Leadership and Operations Centre, USA), Rhonda White (HIV Prevention Trials Network (HPTN) Leadership and Operations Centre, USA), Eric Miller (HIV Prevention Trials Network (HPTN) Leadership and Operations Centre, USA), James Hargreaves (London School of Hygiene & Tropical Medicine, UK), Katharina Hauck (Imperial College, UK), Ranjeeta Thomas (Imperial College, UK), Mohammed Limbada (Zambart, Zambia), Justin Bwalya (Zambart, Zambia), Michael Pickles (Imperial College, UK), Kalpana Sabapathy (London School of Hygiene & Tropical Medicine, UK), Ab Schaap (Zambart, Zambia; and London School of Hygiene & Tropical Medicine, UK), Rory Dunbar (University of Stellenbosch, South Africa), Kwame Shanaube (Zambart, Zambia), Blia Yang (University of Stellenbosch, South Africa), Musonda Simwinga (Zambart, Zambia), Peter Smith (Imperial College, UK), Sten Vermund (HPTN Executive committee), Nomtha Mandla (University of Stellenbosch, South Africa), Nozizwe Makola (University of Stellenbosch, South Africa), Anneen van Deventer (University of Stellenbosch, South Africa), Anelet James (University of Stellenbosch, South Africa), Karen Jennings (City Health Department, Cape Town, South Africa), James Kruger (Department of Health, Western Cape), Mwelwa Phiri (Zambart, Zambia), Barry Kosloff (Zambart, Zambia; and London School of Hygiene & Tropical Medicine, UK), Lawrence Mwenge (Zambart, Zambia), Sarah Kanema (Zambart, Zambia), Rafael Sauter (University of Oxford, UK), William Probert (University of Oxford, UK), Ramya Kumar (Zambart, Zambia; and London School of Hygiene & Tropical Medicine, UK), Ephraim Sakala (Zambart, Zambia), Andrew Silumesi (Ministry of Health, Zambia), Tim Skalland (HPTN Statistical and Data management centre (SDMC), USA), Krista Yuhas (HPTN Statistical and Data management centre (SDMC), USA).
